# Treatment Adherence, Psychosocial Factors, and Clinical Outcomes in Repeatedly Hospitalized Patients with Rheumatoid Arthritis and Ankylosing Spondylitis: An Exploratory Mid-Term Longitudinal Mixed-Effects Study

**DOI:** 10.3390/medsci14020278

**Published:** 2026-05-30

**Authors:** Gabriela Isabela Verga Răuță, Mariana Șerban (Grădinaru), Gabriela Gurău, Carmen Loredana Petrea (Cliveți), Mădălina Nicoleta Matei, Alexia Anastasia Ștefania Baltă, Diana-Andreea Ciortea, Doina Carina Voinescu

**Affiliations:** 1 Faculty of Medicine and Pharmacy, Research Center in the Medico-Pharmaceutical Field, “Dunarea de Jos” University of Galati, 800008 Galati, Romania; carmen.petrea@ugal.ro (C.L.P.); madalina.matei@ugal.ro (M.N.M.); alexiabalta@yahoo.ro (A.A.Ș.B.); diana.ciortea@ugal.ro (D.-A.C.); doina.voinescu@ugal.ro (D.C.V.); 2 ‘Sf. Ioan’ Emergency Clinical Hospital for Children, 800487 Galati, Romania; 3 St. “Apostol Andrei” County Emergency Clinical Hospital, 800578 Galati, Romania; 4 “Maria Sklodowska Curie” Emergency Clinical Hospital for Children, 041451 Bucharest, Romania

**Keywords:** rheumatoid arthritis, ankylosing spondylitis, treatment adherence, disease activity, quality of life, mixed-effects models, psychosocial factors, healthcare utilization

## Abstract

Background: Rheumatoid arthritis (RA) and ankylosing spondylitis (AS) are chronic inflammatory rheumatic diseases associated with impaired quality of life, persistent disease burden, and increased healthcare utilization. Treatment adherence and psychosocial factors may influence outcomes, but their longitudinal associations in real-world hospitalized populations remain insufficiently characterized. Methods: We conducted a single-center, retrospective, mid-term longitudinal observational study including 50 adults with RA or AS who experienced repeated hospitalizations over a four-year period. The final dataset comprised 196 hospitalization episodes analyzed as repeated observations nested within individual patients. Disease activity was assessed using DAS28 in RA and ASDAS and/or BASDAI in AS, according to data availability and routine clinical practice. Treatment adherence, quality of life, anxiety, social isolation, patient–provider communication, dietary support, inflammatory markers, and hospitalization-related outcomes were extracted from medical records and structured inpatient assessments. Linear mixed-effects models were used for continuous outcomes, and ordinal mixed-effects models were used for ordered categorical outcomes, with adjustment for age, sex, and time where appropriate. Results: In RA, higher treatment adherence was associated with lower disease activity over time. In AS, comparable associations were not detected, possibly reflecting disease-specific factors, limited variability in adherence, and reduced statistical power in the smaller AS subgroup. Better patient–provider communication was associated with higher adherence and lower anxiety, whereas greater social isolation was associated with poorer quality of life. More favorable dietary support was associated with better adherence, although the magnitude of this association should be interpreted cautiously because of sparse categories and wide confidence intervals. Lower inflammatory burden, particularly lower CRP over time, was associated with lower hospitalization-related costs. Conclusions: In this selected cohort of repeatedly hospitalized patients with RA or AS, treatment adherence, psychosocial factors, and supportive care indicators were associated with clinically relevant longitudinal outcomes. The findings support a multidisciplinary, patient-centered approach to inflammatory rheumatic disease care. However, because of the retrospective design, modest sample size, selected inpatient population, non-standardized assessment of several variables, and possible instability of some ordinal model estimates, the results should be interpreted as exploratory and confirmed in larger prospective cohorts.

## 1. Introduction

Rheumatoid arthritis (RA) and ankylosing spondylitis (AS) are chronic immune-mediated inflammatory diseases characterized by persistent inflammation, progressive structural damage, pain, and functional limitation [1]. Beyond their direct clinical manifestations, both conditions are associated with long-term disability, impaired work capacity, diminished social participation, and substantial healthcare costs [2]. Despite major advances in pharmacological treatment, including biologic and targeted synthetic disease-modifying therapies, a considerable proportion of patients continue to experience active disease or incomplete symptom control [3,4].

In recent years, disease management in inflammatory rheumatic disorders has progressively shifted from a purely biomedical model toward a broader, patient-centered approach. While control of inflammation remains a central therapeutic objective, growing evidence suggests that biological measures alone do not fully explain the variability observed in clinical response, quality of life, or healthcare utilization [5]. Patient outcomes are increasingly understood as the result of interactions between disease activity, treatment-related factors, behavioral patterns, and psychosocial context [6].

Among these factors, treatment adherence plays a particularly important role. In chronic diseases requiring sustained long-term therapy, adherence represents the practical link between the efficacy demonstrated in clinical trials and the effectiveness achieved in routine care [7]. In inflammatory arthritis, suboptimal adherence has been associated with poorer disease control, increased symptom burden, and reduced treatment benefit [8]. However, adherence is not a static behavior; rather, it is shaped by multiple individual and contextual influences, including patient beliefs, perceived treatment burden, emotional distress, health literacy, and the quality of therapeutic relationships [9,10].

Psychosocial variables are especially relevant in this context. Anxiety, social isolation, inadequate family support, and sleep disturbances may negatively affect self-management capacity and engagement with care, thereby worsening both subjective and objective outcomes [11,12]. Conversely, effective communication with healthcare professionals may improve trust, shared decision-making, and understanding of treatment goals, which may in turn support better adherence and higher satisfaction with care [13]. These relationships are clinically important because they suggest that patient outcomes may be improved not only through pharmacological optimization, but also through structured psychosocial and educational support.

Supportive and nursing-related interventions have therefore received increasing attention in chronic rheumatic disease management. Patient education, dietary counseling, continuity of care, and repeated therapeutic reinforcement may enhance self-management and facilitate behavior change [14,15]. Nevertheless, the effect of such interventions is often indirect and may operate through complex pathways involving adherence, emotional well-being, and perceived disease burden. These mechanisms remain insufficiently explored in longitudinal clinical datasets.

Most previous studies addressing adherence, psychosocial factors, or patient-reported outcomes in inflammatory rheumatic diseases have been cross-sectional or have examined only one domain at a time [16,17,18]. As a result, the temporal relationships among disease activity, adherence, quality of life, and healthcare utilization remain incompletely understood. Comparative evidence between RA and AS is particularly limited, despite important differences in pathophysiology, demographics, symptom profiles, and therapeutic management [19,20].

Longitudinal mixed-effects modeling provides a robust framework for investigating these issues because it allows repeated observations to be analyzed while accounting for both within-patient change and between-patient variability [21,22]. Such models are especially appropriate in real-world clinical studies where outcomes are measured across multiple hospitalizations or follow-up encounters and where the contribution of behavioral and psychosocial variables may evolve over time.

Accordingly, the aim of the present study was to examine the longitudinal associations of treatment adherence, psychosocial factors, supportive care variables, disease activity, quality of life, and healthcare utilization in patients with rheumatoid arthritis and ankylosing spondylitis. By integrating clinical, behavioral, and psychosocial domains within a unified analytical framework, this study seeks to provide a more comprehensive understanding of the factors associated with patient outcomes in inflammatory rheumatic disease and to identify clinically relevant targets for multidisciplinary care.

## 2. Materials and Methods

### 2.1. Study Design

This study was designed as a single-center, retrospective, mid-term longitudinal observational cohort study evaluating associations among treatment adherence, psychosocial factors, disease activity, quality of life, inflammatory markers, and healthcare utilization in patients with rheumatoid arthritis (RA) and ankylosing spondylitis (AS).

The study was conducted in a tertiary rheumatology care setting at St. Apostle Andrew Clinical Emergency County Hospital, Galați, Romania, between 1 January 2022 and 30 October 2025. Data were collected from routine inpatient care and structured clinical assessments documented during hospitalization. No experimental intervention was introduced for research purposes, and all patients received standard-of-care management according to contemporary rheumatology practice and institutional protocols.

Because the dataset included repeated hospitalizations for the same patients, the unit of longitudinal observation was the hospitalization episode. Repeated hospitalization episodes were nested within individual patients and analyzed using mixed-effects models with patient-specific random intercepts. Thus, the analytical structure allowed within-patient correlation to be taken into account while also capturing between-patient variability.

The four-year observation period should be interpreted as a mid-term follow-up interval rather than a long-term study. The analytical time points T0, T12, T24, and T36 represented approximate 12-month follow-up windows based on available hospitalization episodes and should not be interpreted as protocol-mandated visits attended by all patients at exact predefined dates.

### 2.2. Study Population

The study population consisted of 50 adult patients diagnosed with either rheumatoid arthritis or ankylosing spondylitis and followed longitudinally throughout the study period. Patients were eligible for inclusion if they met all of the following criteria:

(1) Age ≥18 years;
(2) Confirmed diagnosis of RA or AS according to accepted classification criteria;
(3) At least four recorded hospitalizations during the study period;
(4) Availability of clinical and psychosocial data required for longitudinal analysis.

RA was classified according to the 2010 American College of Rheumatology/European Alliance of Associations for Rheumatology (ACR/EULAR) classification criteria, whereas ankylosing spondylitis was classified according to the modified New York criteria.

Exclusion criteria were:

(1) Missing key clinical data required for the main analyses;
(2) Severe cognitive impairment or major psychiatric disorder interfering with reliable self-reporting;
(3) Major concomitant medical conditions judged by the investigators to substantially confound the evaluation of disease activity or quality of life.

The final analytical dataset comprised 196 hospitalization episodes, representing repeated measurements nested within the 50 included patients. Of these, 30 patients had RA and 20 had AS. Demographic variables, including age and sex, were recorded at baseline and included as covariates in adjusted models.

### 2.3. Clinical Characterization of the Cohort

Where available, additional clinical variables were extracted from medical records to improve characterization of the study population. These included disease duration before inclusion, serological status in RA, including rheumatoid factor and anti-citrullinated protein antibodies, HLA-B27 status in AS, relevant comorbidities, previous and current use of conventional synthetic DMARDs, biologic DMARDs, targeted synthetic DMARDs, non-steroidal anti-inflammatory drugs, and glucocorticoids. Treatment modifications during follow-up were also recorded when clearly documented in the medical record.

Previous exposure to psychoeducational interventions or structured self-management education was recorded when available. Because the study was retrospective and based on routine inpatient documentation, some of these variables were incompletely available. Therefore, they were used primarily for descriptive characterization rather than as covariates in all longitudinal models.

The main reason for each hospitalization was classified, where possible, into clinically meaningful categories, including disease activity assessment, treatment optimization, flare management, rehabilitation or supportive care, comorbidity-related admission, or other reasons. This information was used to contextualize hospitalization frequency, length of stay, and hospitalization-related costs.

#### 2.3.1. Disease Activity

Disease activity was assessed using disease-specific composite indices documented during routine inpatient evaluation. In patients with RA, disease activity was evaluated using DAS28. In patients with AS, disease activity was evaluated using ASDAS and/or BASDAI, depending on the availability of the required clinical and laboratory components in the medical record.

For AS, ASDAS was considered the preferred composite index when both patient-reported components and inflammatory markers were available. BASDAI was used as a patient-reported disease activity measure and as a complementary or alternative index when ASDAS components were incomplete. Disease activity scores were analyzed as continuous variables in the main longitudinal models. Where clinically meaningful, established severity categories were used for descriptive or ordinal analyses.

Because disease activity measures differ between RA and AS, disease-specific models were fitted separately for DAS28 in RA and for ASDAS/BASDAI in AS.

#### 2.3.2. Treatment Adherence

Treatment adherence was assessed at each hospitalization as an ordinal clinical variable with five levels: complete adherence, good adherence, moderate adherence, low adherence, and no adherence. Because the study was based on routine inpatient practice, adherence was not measured using a validated questionnaire such as the Morisky Medication Adherence Scale. Instead, adherence categories were assigned during hospitalization by the clinical team based on a pragmatic assessment combining patient self-report, medication history, consistency with prescribed treatment, documentation in the medical record, and, when available, information from previous admissions.

Complete adherence indicated that the patient reported following the prescribed therapeutic regimen without relevant omissions. Good adherence indicated only occasional missed doses or minor deviations from the treatment plan. Moderate adherence indicated intermittent omissions or partial implementation of the prescribed regimen. Low adherence indicated frequent omissions, delayed administration, or repeated deviations from the recommended treatment. No adherence indicated discontinuation, refusal, or absence of regular use of the prescribed therapy.

This adherence variable should therefore be interpreted as a real-world clinical estimate of treatment-taking behavior rather than as a validated psychometric measure. Its use improves the pragmatic relevance of the study but limits reproducibility and comparability with studies using standardized adherence instruments.

#### 2.3.3. Quality of Life

Quality of life was assessed at each hospitalization as a patient-reported variable categorized into four ordered levels: excellent, good, moderate, and poor/very poor. This measure reflected the patient’s global perception of well-being and functional status as recorded during inpatient assessment. For descriptive purposes, quality of life was treated as an ordered categorical variable. In longitudinal analyses, it was modeled either as an approximately continuous ordered score or as an ordinal outcome, depending on the specific analytical objective and the need to preserve category structure.

#### 2.3.4. Psychosocial Variables

Psychosocial variables were recorded at each hospitalization using ordinal categories documented in routine clinical evaluation. These included:

- Family support: excellent, good, moderate, or low.
- Patient–provider communication: excellent, good, moderate, or difficult.
- Anxiety: absent, mild, moderate, severe, or extreme.
- Sleep disturbances: normal sleep, mild disturbance, moderate disturbance, severe disturbance, or marked insomnia.
- Social isolation: none, minimal, moderate, severe, or total.

These variables were included in the analysis as indicators of psychosocial burden and interpersonal support in the clinical care context.

#### 2.3.5. Socioeconomic Variables

- Socioeconomic characteristics included:
- Educational level, categorized into ordinal levels from lower to higher educational attainment.
- Occupational status, categorized as active employment, disability pension, unemployed, or other.

These variables were considered potential contextual factors associated with treatment adherence, disease activity, and patient-reported outcomes.

#### 2.3.6. Biological Parameters

Inflammatory activity was additionally assessed using routinely collected laboratory markers:

- C-reactive protein (CRP);
- Erythrocyte sedimentation rate (ESR).

Both variables were treated as continuous measures. They were included in the analysis as markers of inflammatory burden and were also examined in relation to hospitalization-related costs.

#### 2.3.7. Supportive Care Variables

To capture the contribution of supportive care, the following variables were included:

- Dietary support, categorized as complete and adhered, good, moderate, low, or absent. In this study, dietary support reflected the level of diet-related counseling and support documented during inpatient care together with the extent to which dietary recommendations were followed, as captured in routine clinical assessment.
- Patient–provider communication, which was analyzed as a psychosocial care-process variable and interpreted as reflecting both interpersonal quality and supportive care during hospitalization.

#### 2.3.8. Healthcare Utilization Outcomes

Healthcare utilization variables included:

- Number of hospitalizations during the study period;
- Length of hospitalization, recorded in days;
- Hospitalization costs, expressed in Romanian currency (RON) per admission.

### 2.4. Outcome Definitions

The main outcomes of interest were:

- Disease activity, assessed continuously using DAS28, ASDAS, or BASDAI depending on diagnosis;
- Treatment adherence, modeled as an ordinal longitudinal outcome;
- Quality of life, assessed as an ordered patient-reported variable and modeled either as an approximately continuous longitudinal outcome in selected analyses or as an ordered categorical outcome when preservation of ordinal structure was considered more appropriate;
- Healthcare utilization, assessed through hospitalization duration and hospitalization costs.

Secondary outcomes included psychosocial variables, particularly anxiety, and the evaluation of associations between supportive care indicators and patient-reported outcomes.

### 2.5. Statistical Analysis

#### 2.5.1. Descriptive Analysis

Descriptive statistics were used to summarize baseline patient characteristics and repeated-measure variables. Continuous variables were described using means and standard deviations (SD) or medians and interquartile ranges (IQR), depending on their distribution. Categorical variables were expressed as frequencies and percentages.

Comparisons between diagnostic groups were performed using appropriate parametric or non-parametric methods according to data type and distribution.

#### 2.5.2. Longitudinal Modeling Strategy

Because the data had a hierarchical longitudinal structure, with repeated hospitalizations nested within patients, mixed-effects models were used to account for within-patient correlation and between-patient heterogeneity. For continuous outcomes, including DAS28, ASDAS, BASDAI, CRP, ESR, length of hospitalization, and log-transformed hospitalization costs, linear mixed-effects models (LMMs) with random intercepts for patient were fitted. For ordered categorical outcomes, including treatment adherence, anxiety, social isolation, family support, patient–provider communication, dietary support, and, where applicable, quality of life, ordinal mixed-effects models with cumulative logit link functions were fitted.

Quality of life was recorded as a four-level ordered variable (excellent, good, moderate, and poor/very poor). In analyses examining overall longitudinal trends and selected associations, it was treated as an approximately continuous outcome and analyzed using linear mixed-effects models as a pragmatic summary measure. However, in analyses where preservation of the ordinal structure was considered more appropriate—specifically the model examining the association between social isolation and quality of life—it was analyzed as an ordered categorical outcome using an ordinal mixed-effects model with a cumulative logit link. Accordingly, associations involving quality of life are reported either as linear mixed-model estimates or as odds ratios, depending on the modeling approach used in the specific analysis.

Disease activity outcomes were modeled separately by diagnosis because different validated instruments were used in RA and AS. Specifically, DAS28 models were fitted in the RA subgroup, whereas ASDAS and BASDAI models were fitted in the AS subgroup.

#### 2.5.3. Covariates and Model Adjustment

The main predictors entered into the models included treatment adherence, psychosocial factors, supportive care variables, inflammatory markers, and diagnostic group where appropriate. All adjusted models included age, sex, and time as covariates. Time was coded as annual admissions coded at 12-month intervals over a 4-year period (T0, T12, T24, T36).

Where clinically justified, disease-specific analyses were performed separately for RA and AS, especially for outcomes based on disease-specific activity indices. When relevant, interaction terms such as time × predictor were examined to assess whether the association between a predictor and outcome changed over follow-up; these interaction analyses were considered exploratory.

#### 2.5.4. Model Diagnostics and Reporting

Model assumptions were assessed using residual diagnostics and goodness-of-fit procedures for linear mixed-effects models. Because hospitalization costs showed marked right skewness, log transformation was used to improve model fit and approximate residual normality.

Effect estimates were reported as:

- β coefficients with standard errors (SEs) or 95% confidence intervals (CIs) for linear mixed-effects models;
- F statistics and corresponding *p*-values for fixed effects in linear mixed-effects models where appropriate;
- Odds ratios (ORs) with 95% CIs for ordinal mixed-effects models.
- Given the modest sample size and the exploratory nature of several analyses, effect sizes and confidence intervals were prioritized where available, and findings derived from pragmatic modeling choices were interpreted cautiously.

Given the modest number of unique patients and the relatively large number of candidate predictors, the statistical analyses were considered exploratory. The repeated hospitalization episodes increased the number of observations but did not remove the limitation imposed by the small number of individual patients. Therefore, subgroup analyses, interaction terms, and ordinal models with sparse categories were interpreted cautiously.

Very large odds ratios obtained from ordinal mixed-effects models were not interpreted as precise estimates of effect magnitude. In particular, when some outcome or predictor categories contained few observations, estimates may have been unstable and confidence intervals inflated. In such cases, emphasis was placed on the direction and consistency of the association rather than on the exact numerical value of the odds ratio.

#### 2.5.5. Missing Data

Missing data were addressed using a complete-case approach applied separately to each model. Hospitalization episodes with missing values for the dependent variable or any covariate included in a given analysis were excluded from that specific model only. As a result, the effective sample size differed across analyses.

No formal multiple imputation or sensitivity analyses were undertaken. Given that the dataset was derived from routine clinical practice, the assumption that data were missing completely at random may not hold. Accordingly, some degree of bias due to missing data cannot be ruled out.

#### 2.5.6. Statistical Software

All analyses were performed using IBM SPSS version 27 and Excel 2019 MSO (Version 2503). Linear mixed-effects models were fitted using the MIXED procedure. Ordinal mixed-effects models were fitted using the GENLINMIXED procedure with a cumulative logit link and random intercept for patient.

### 2.6. Ethical Considerations

The study was conducted in accordance with the principles of the Declaration of Helsinki and was approved by the Ethics Committee of the “Dunărea de Jos” University in Galați under approval number CEU 77/12.12.2025. Written informed consent was obtained from each patient at the time of admission, including consent for the subsequent use of medical data routinely collected during the examinations.

All data were anonymized prior to analysis, and patient confidentiality was maintained throughout the study.

## 3. Results

### 3.1. Study Population Characteristics

The study included 50 patients, of whom 30 had rheumatoid arthritis (RA) and 20 had ankylosing spondylitis (AS). These patients contributed a total of 196 hospitalization episodes, including 117 admissions in the RA group and 79 admissions in the AS group.

The mean age of the overall cohort was 54.14 ± 11.59 years. Patients with RA were significantly older than those with AS (*p* = 0.001). Sex distribution differed significantly between diagnostic groups (*p* < 0.001), with women predominating in the RA group (83.33%) and men predominating in the AS group (75.00%).

The overall mean length of hospitalization was 5.37 ± 4.26 days. Hospital stays were significantly longer in patients with RA than in those with AS (*p* = 0.019). The mean number of hospitalizations per patient during follow-up was 3.92 overall, corresponding to 3.90 in RA and 3.95 in AS.

Baseline demographic and hospitalization characteristics are summarized in Table 1.

Additional clinical characteristics are presented in Table 2. Disease duration was available for 46/50 patients (92.0%), with a median duration of 10.5 years and mean duration of 11.4 ± 6.8 years. Among RA patients, 22/28 (78.6%) were RF-positive and 20/27 (74.1%) were ACPA-positive. Among AS patients, 16/18 (88.9%) were HLA-B27-positive. Comorbidities were present in 33/50 patients (66.0%), more frequently in RA than AS (76.7% vs. 50.0%). csDMARD use was higher in RA (93.3% vs. 40.0%), whereas NSAID use predominated in AS (90.0% vs. 53.3%). Treatment modification occurred in 29/50 patients (58.0%).

Values are presented as n (%) unless otherwise specified. Disease duration is reported among patients with available data. Main hospitalization reason is reported at the admission level, whereas the other variables are reported at the patient level.

Main reasons for hospitalization are presented in Table 3. All 196 admissions had available data. The most frequent reason was disease flare or high disease activity, accounting for 62/196 admissions (31.6%), more often in RA than AS (36.8% vs. 24.1%). Treatment optimization or clinical monitoring represented 48/196 admissions (24.5%). Biologic or targeted therapy assessment/administration accounted for 35/196 admissions (17.9%), with similar proportions in AS and RA. Rehabilitation, supportive care, or pain management represented 26/196 admissions (13.3%) and was more frequent in AS (17.7%) than RA (10.3%). Comorbidity, infection, or laboratory monitoring accounted for 17/196 admissions (8.7%).

### 3.2. Distribution of Treatment Adherence

Treatment adherence was generally high across hospitalization episodes. Complete adherence was observed in 45.92% of episodes and good adherence in 36.73%, indicating that most observations fell within the favorable adherence range. Moderate adherence accounted for 11.22% of episodes, whereas low adherence (5.10%) and no adherence (1.02%) were infrequent. These results suggest an overall high level of recorded adherence in the study cohort. These data are presented in Table 4.

### 3.3. Association Between Adherence and Disease Activity

In diagnosis-specific longitudinal analyses, treatment adherence was significantly associated with disease activity in the RA group. Higher adherence was associated with lower DAS28 values over time (F=11.702, p<0.001). Estimated marginal means showed a gradient across adherence categories, with lower DAS28 values observed among patients with complete adherence and higher values in lower-adherence categories.

However, these category-specific estimates should be interpreted cautiously because the extreme adherence categories were sparsely represented, particularly the “no adherence” category, which included only two hospitalization episodes in the overall dataset. Accordingly, non-monotonicity at the lowest adherence levels is likely to reflect limited precision rather than a clinically meaningful reversal of the overall trend.

In the AS group, treatment adherence was not significantly associated with disease activity assessed either by ASDAS (p=0.944) or BASDAI (p=0.145).

These findings indicate that the longitudinal association between adherence and inflammatory disease control was more evident in RA than in AS (Table 5).

### 3.4. Association Between Treatment Adherence and Quality of Life

Quality of life was originally recorded as an ordered four-level patient-reported variable. For the analyses presented in this section, it was treated as an approximately continuous ordered score in linear mixed-effects models to evaluate overall longitudinal trends and associations. In contrast, when the objective was to preserve the full ordinal structure of the variable, such as in the analysis of social isolation and quality-of-life categories, ordinal mixed-effects models were used. Therefore, results involving quality of life are reported either as F statistics from linear mixed-effects models or as odds ratios from ordinal mixed-effects models, depending on the specific analytical approach.

In patients with RA, higher treatment adherence was significantly associated with better quality of life in the unadjusted longitudinal model (F = 4.584, *p* = 0.002). However, after including disease activity in the model, the association was attenuated and no longer statistically significant (*p* = 0.130), suggesting that differences in disease activity may partly explain the association between adherence and quality of life.

In the AS group, treatment adherence was not significantly associated with quality of life (*p* = 0.220).

These findings indicate that the association between adherence and quality of life was more evident in RA than in AS and appeared to be partly accounted for by inflammatory disease control (Table 6).

### 3.5. Dietary Support and Treatment Adherence

Dietary support was associated with treatment adherence in the ordinal mixed-effects model. Patients with more favorable dietary support had higher odds of being classified in a better adherence category over time (OR = 68.64, 95% CI: 8.45–557.84, *p* < 0.001). However, the magnitude of this odds ratio should be interpreted with caution because the confidence interval was wide and some adherence categories were sparsely represented. Therefore, this finding should be interpreted as evidence of a positive association between dietary/supportive care engagement and adherence, rather than as a precise estimate of effect size. No significant associations were observed for age or sex (Table 7).

### 3.6. Socioeconomic Factors and Disease Activity

Educational level and occupational status were not significantly associated with treatment adherence in ordinal mixed-effects analyses (education: p=0.827; occupation: p=0.762).

However, occupational status was significantly associated with disease activity. Professionally active patients had lower disease activity scores than non-active patients in both RA and AS. In RA, occupational status was associated with DAS28 (F=7.056, p=0.001), whereas in AS it was associated with ASDAS (F=3.263, p=0.026).

These results suggest that socioeconomic functioning, particularly work participation, may be associated with lower disease burden, although the direction of this relationship cannot be determined in an observational design (Table 8).

### 3.7. Social Isolation and Quality of Life

Most patients reported minimal or moderate social isolation. No significant differences in social isolation were observed between RA and AS (*p* = 0.307), and no significant change over time was identified (*p* = 0.610). Nevertheless, higher social isolation was strongly associated with worse quality-of-life categories in the ordinal mixed-effects model (OR = 45.24, 95% CI: 17.71–115.56, *p* < 0.001).

Although the direction of this association is clinically plausible, the magnitude of the odds ratio should be interpreted cautiously. The modest sample size and sparse observations in extreme categories may have contributed to unstable or inflated estimates. Therefore, this result should be understood primarily as indicating that greater social isolation clustered with poorer quality of life, rather than as a precise quantification of risk (Table 9).

### 3.8. Patient–Provider Communication, Anxiety, and Adherence

Patient–provider communication emerged as an important longitudinal correlate of psychosocial outcomes. Better patient–provider communication was significantly associated with better adherence categories (OR = 2.84, 95% CI: 1.71–4.73, *p* < 0.001), whereas greater anxiety severity was associated with poorer adherence categories (OR = 6.40, 95% CI: 2.08–19.65, *p* = 0.001). These results are summarized in Table 10.

### 3.9. Supportive Care and Hospitalization Outcomes

Supportive care indicators were also associated with hospitalization-related outcomes. Better patient–provider communication was associated with shorter hospitalization duration (F=3.215, p=0.024). Similarly, more favorable dietary support was associated with shorter hospital stays (F=3.766, p=0.006). Treatment adherence showed a similar trend toward shorter hospitalization duration, although this did not reach statistical significance (F=2.092, p=0.084). These results should be interpreted as associative rather than causal, given the observational design and the pragmatic definition of supportive care variables. These results are shown in Table 11.

### 3.10. Disease Activity, Inflammation, and Hospitalization Costs

Hospitalization cost analyses were conducted using log-transformed cost data. Cost data were available for all RA admissions. DAS28 was not significantly associated with hospitalization costs (p=0.140). In contrast, higher CRP levels were significantly associated with higher hospitalization costs (β=0.06, F=5.953, p=0.020). In addition, the interaction between time and CRP was statistically significant (β=−0.001, F=5.531, p=0.025), indicating that decreases in inflammatory burden over time were associated with lower hospitalization-related costs. Age was also a significant positive predictor of hospitalization costs (p<0.05). These findings suggest that inflammatory burden may be more closely associated with economic outcomes than composite disease activity scores alone, although the cost analyses should be interpreted cautiously given the limited sample and possible restriction of cost data to a subset of admissions. These findings are summarized in Table 12.

## 4. Discussion

### 4.1. Principal Findings

This longitudinal study examined the associations among treatment adherence, disease activity, quality of life, psychosocial variables, and healthcare utilization in patients with rheumatoid arthritis (RA) and ankylosing spondylitis (AS). The findings suggest that treatment adherence is an important correlate of longitudinal outcomes, particularly in RA, where higher adherence was associated with lower disease activity and better quality of life. The attenuation of the adherence–quality of life association after adjustment for DAS28 is consistent with the possibility that disease control partly accounts for this relationship [17,18,19].

In AS, adherence was not significantly associated with disease activity or quality of life in the present dataset. This difference may reflect disease-specific mechanisms, limited variability in adherence, or reduced statistical power in subgroup analyses rather than a true absence of association [20].

### 4.2. A Biopsychosocial Framework of Disease Outcomes

The results are consistent with a multidimensional biopsychosocial framework in which clinical outcomes are shaped by interactions among biological, behavioral, and psychosocial factors. Within this framework, treatment adherence may function as an intermediary behavioral variable linking upstream determinants—such as communication, anxiety, and supportive care—to downstream outcomes including disease activity and quality of life.

The strong association between social isolation and poorer quality of life highlights the relevance of non-biological determinants in chronic inflammatory diseases. However, given the magnitude of the observed odds ratio and the modest sample size, the numerical estimate should be interpreted cautiously, as sparse data in some categories may have inflated the effect size [23].

### 4.3. Differential Patterns in RA and AS

A notable finding of this study is the apparent difference between RA and AS regarding the association between adherence and disease activity. In RA, higher adherence was associated with lower DAS28 values, whereas in AS no statistically significant association was detected for ASDAS or BASDAI. However, this contrast should be interpreted cautiously. The AS subgroup included fewer patients than the RA subgroup, and the number of observations may have been insufficient to detect moderate associations, particularly if adherence variability was limited. Therefore, the absence of statistically significant associations in AS should not be interpreted as evidence that adherence is clinically unimportant in AS.

First, AS outcomes may be influenced not only by inflammatory activity but also by structural damage, pain processing, and biomechanical limitation, which may reduce the apparent short-term relationship between pharmacological adherence and disease activity indices. Second, adherence was generally high among AS patients, which may have limited variability and constrained the ability to detect associations. Third, non-pharmacological components of management, such as exercise and rehabilitation, may play a comparatively stronger role in AS outcomes [24,25,26].

### 4.4. Psychosocial Determinants of Outcomes

Psychosocial variables emerged as important correlates of patient outcomes. Better patient–provider communication was associated with both higher adherence and lower anxiety severity, supporting prior evidence that communication quality may facilitate treatment engagement, understanding of care plans, and emotional reassurance [27,28].

Social isolation was strongly associated with poorer quality of life, while anxiety was associated with poorer adherence. Together, these findings reinforce the clinical relevance of psychosocial assessment in chronic inflammatory rheumatic disease management [29,30,31].

### 4.5. Supportive Care and Behavioral Outcomes

Supportive care variables, particularly dietary support, were associated with both adherence and quality of life. In interpreting these results, it is important to note that dietary support in this study was a pragmatic clinical variable rather than a standardized nutritional intervention. Therefore, the observed associations should not be interpreted as evidence of a specific causal dietary effect, but rather as suggesting that stronger supportive care and better engagement with recommendations may cluster with more favorable patient-reported outcomes [13,31,32].

Patient–provider communication was analyzed as a psychosocial care-process variable and may reasonably be understood as occupying both psychosocial and supportive-care domains. This dual interpretation is clinically plausible, although it should be recognized conceptually when interpreting the models.

### 4.6. Healthcare Utilization and Economic Implications

Disease activity assessed by DAS28 was not significantly associated with hospitalization costs in the present analysis. By contrast, higher CRP and the time × CRP interaction were associated with cost trajectories, suggesting that inflammatory burden may have a closer relationship to hospitalization-related expenditures than composite disease activity scores in this dataset.

These findings should be interpreted carefully because cost data may have been available only for a subset of admissions, and the economic analysis was conducted in a relatively small observational sample.

## 5. Limitations

Several limitations should be acknowledged when interpreting these findings.

First, this was a single-center retrospective observational study including a modest number of unique patients. Although the dataset included 196 hospitalization episodes, repeated admissions from the same patients do not fully compensate for the limited number of individuals. This is particularly relevant for subgroup analyses in AS, where the smaller sample size may have reduced statistical power to detect significant associations.

Second, the cohort was composed of patients with repeated hospitalizations and therefore represents a selected inpatient population. These patients may differ from the broader RA and AS populations managed primarily in outpatient settings. Consequently, selection bias is possible, and the findings should not be generalized to all patients with RA or AS.

Third, several important rheumatological variables were unavailable or incompletely documented in routine medical records. These include disease duration, disease phenotype, RF and ACPA status in all RA patients, HLA-B27 status in all AS patients, detailed comorbidity profiles, previous treatment history, cumulative glucocorticoid exposure, biologic or targeted synthetic DMARD use, and treatment changes over time. These variables may confound the associations among adherence, disease activity, quality of life, and hospitalization outcomes.

Fourth, treatment adherence, quality of life, anxiety, social isolation, patient–provider communication, and dietary support were assessed using pragmatic clinical categories rather than standardized validated instruments. Although this reflects real-world inpatient practice, it limits reproducibility, comparability with other studies, and precision of measurement. Future studies should use validated tools such as standardized adherence scales, health-related quality-of-life questionnaires, and validated measures of anxiety and social support.

Fifth, the reasons for hospitalization were not uniformly available for all admissions. This limits interpretation of hospitalization frequency, length of stay, and cost-related outcomes. In contemporary rheumatology care, repeated hospitalizations may reflect disease severity, treatment optimization, comorbidity burden, institutional practice patterns, or social factors; these possibilities could not be fully disentangled in the present dataset.

Sixth, the statistical models should be considered exploratory. The number of predictors, subgroup analyses, and interaction terms was relatively large compared with the number of unique patients. Some ordinal categories were sparsely populated, which may have resulted in unstable estimates and very large odds ratios with wide confidence intervals. For this reason, the direction of associations may be more reliable than the exact magnitude of the effect estimates.

Seventh, missing data were handled using model-specific complete-case analysis. Because data were derived from routine clinical documentation, missingness may not have been completely random, and residual bias cannot be excluded.

Finally, the observational design precludes causal inference. The associations observed among adherence, psychosocial factors, supportive care indicators, disease activity, and healthcare utilization should be interpreted as exploratory and hypothesis-generating rather than causal.

## 6. Future Directions

Future studies should aim to validate these findings in larger, multicenter cohorts with more balanced subgroup sizes and more precise measurement of psychosocial and behavioral constructs. The use of standardized validated instruments for adherence, quality of life, communication, anxiety, and social isolation would improve comparability and reduce measurement error.

In addition, future research should incorporate fuller reporting of mixed-model parameters, including fixed-effect estimates, confidence intervals, and variance components, to strengthen interpretability and reproducibility. Objective adherence measures, such as pharmacy refill data or digital monitoring, may also help clarify the role of treatment-taking behavior in longitudinal disease outcomes.

Finally, prospective studies using mediation analysis or structural equation modeling may better disentangle the relationships among adherence, psychosocial factors, inflammatory control, quality of life, and healthcare utilization.

## 7. Conclusions

In this selected cohort of repeatedly hospitalized patients with rheumatoid arthritis or ankylosing spondylitis, treatment adherence was associated with more favorable longitudinal outcomes, particularly in RA, where higher adherence was related to lower disease activity and better quality of life. In AS, similar associations were not statistically significant, possibly because of disease-specific factors, limited adherence variability, and reduced statistical power in the smaller subgroup.

Psychosocial and supportive care factors, including patient–provider communication, dietary support, social isolation, and anxiety, were also associated with patient-reported and hospitalization-related outcomes. These findings are consistent with a multidimensional, patient-centered approach to inflammatory rheumatic disease care.

However, because of the retrospective design, selected inpatient population, modest sample size, non-standardized assessment of several variables, and possible instability of some model estimates, the findings should be interpreted as exploratory. Larger prospective studies using validated instruments and more detailed clinical characterization are needed to confirm these associations.

## Figures and Tables

**Table 1:** Baseline Characteristics of the Study Population.

Variable	Total (n = 50)	RA (n = 30)	AS (n = 20)	*p*-Value
Age (years), mean ± SD	54.14 ± 11.59	58.90 ± 9.84	47.00 ± 10.21	0.001
Female sex, n (%)	30 (60.00)	25 (83.33)	5 (25.00)	<0.001
Male sex, n (%)	20 (40.00)	5 (16.67)	15 (75.00)	<0.001
Patients, n	50	30	20	—
Total hospitalizations, n	196	117	79	—
Hospitalizations per patient, mean	3.92	3.90	3.95	0.918
Length of hospital stay (days), mean ± SD	5.37 ± 4.26	6.41 ± 4.40	3.81 ± 3.30	0.019

**Table 2:** Additional Clinical Characteristics of the Study Population.

Variable	Total	RA	AS	Available Data, n (%)
Disease duration, years, mean ± SD/median (IQR)	11.4 ± 6.8/10.5 (6.0–16.0)	10.8 ± 6.2/9.5 (5.0–15.0)	12.3 ± 7.1/11.0 (7.0–18.0)	46/50 (92.0)
RF-positive, n (%)	—	22/28 (78.6)	—	28/30 RA patients (93.3)
ACPA-positive, n (%)	—	20/27 (74.1)	—	27/30 RA patients (90.0)
HLA-B27-positive, n (%)	—	—	16/18 (88.9)	18/20 AS patients (90.0)
≥1 comorbidity, n (%)	33/50 (66.0)	23/30 (76.7)	10/20 (50.0)	50/50 (100.0)
Current csDMARD use, n (%)	36/50 (72.0)	28/30 (93.3)	8/20 (40.0)	50/50 (100.0)
Current bDMARD use, n (%)	19/50 (38.0)	10/30 (33.3)	9/20 (45.0)	50/50 (100.0)
Current tsDMARD use, n (%)	3/50 (6.0)	3/30 (10.0)	0/20 (0.0)	50/50 (100.0)
Glucocorticoid exposure, n (%)	25/50 (50.0)	20/30 (66.7)	5/20 (25.0)	50/50 (100.0)
NSAID use, n (%)	34/50 (68.0)	16/30 (53.3)	18/20 (90.0)	50/50 (100.0)
Previous psychoeducational intervention, n (%)	13/50 (26.0)	8/30 (26.7)	5/20 (25.0)	50/50 (100.0)
Treatment modification during follow-up, n (%)	29/50 (58.0)	18/30 (60.0)	11/20 (55.0)	50/50 (100.0)

RA, rheumatoid arthritis; AS, ankylosing spondylitis; RF, rheumatoid factor; ACPA, anti-citrullinated protein antibodies; HLA-B27, human leukocyte antigen B27; csDMARDs, conventional synthetic disease-modifying antirheumatic drugs; bDMARDs, biologic disease-modifying antirheumatic drugs; tsDMARDs, targeted synthetic disease-modifying antirheumatic drugs; NSAIDs, non-steroidal anti-inflammatory drugs.

**Table 3:** Main Reasons for Hospitalization.

Main Hospitalization Reason	Total Admissions, n (%)	RA Admissions, n (%)	AS Admissions, n (%)	Available Data, n (%)
Disease flare/high disease activity	62/196 (31.6)	43/117 (36.8)	19/79 (24.1)	196/196 (100.0)
Treatment optimization/clinical monitoring	48/196 (24.5)	30/117 (25.6)	18/79 (22.8)	196/196 (100.0)
Biologic or targeted therapy assessment/administration	35/196 (17.9)	17/117 (14.5)	18/79 (22.8)	196/196 (100.0)
Rehabilitation, supportive care, or pain management	26/196 (13.3)	12/117 (10.3)	14/79 (17.7)	196/196 (100.0)
Comorbidity, infection, or laboratory monitoring	17/196 (8.7)	11/117 (9.4)	6/79 (7.6)	196/196 (100.0)
Other/administrative reason	8/196 (4.1)	4/117 (3.4)	4/79 (5.1)	196/196 (100.0)

**Table 4:** Distribution of treatment adherence across hospitalization episodes.

Adherence Category	n	%
Complete adherence	90	45.92
Good adherence	72	36.73
Moderate adherence	22	11.22
Low adherence	10	5.10
No adherence	2	1.02
Total	196	100.00

**Table 5:** Adherence and Disease Activity (RA).

Adherence Level	DAS28
Complete	3.99
Good	5.12
Moderate	5.21
Low	6.11
None	5.32

**Note:** The “none” category should be interpreted cautiously because of the very small number of observations.

**Table 6:** Association Between Treatment Adherence and Quality of Life.

Model	F	*p*-Value
RA, unadjusted	4.584	0.002
RA, adjusted for DAS28	—	0.130
AS	—	0.220

**Table 7:** Ordinal Mixed-Effects Model for Treatment Adherence.

Model Term	Coefficient	Std. Error	t	Sig.	95% Confidence Interval	Exp (Coefficient)	95% Confidence Interval for Exp (Coefficient)
					Lower/Upper		Lower/Upper
Threshold for Treatment Adherence = 0.00	3.225	4.1444	0.778	0.438	−4.988 to 11.439	25.164	0.007 to 92,848.108
Threshold for Treatment Adherence = 1.00	10.387	4.4012	2.360	0.020	1.664 to 19.109	32,423.831	5.283 to 198,996,542.789
Threshold for Treatment Adherence = 2.00	15.053	4.7011	3.202	0.002	5.737 to 24.370	3,448,335.000	310.110 to 38,344,462,573.000
Threshold for Treatment Adherence = 3.00	19.715	5.1312	3.842	<0.001	9.546 to 29.883	364,670,438.000	13,983.341 to 9,510,211,335,083.000
Dietary support	4.229	1.0572	4.000	<0.001	2.134 to 6.324	68.643	8.447 to 557.840
Sex = 1	−1.850	1.9255	−0.961	0.339	−5.666 to 1.966	0.157	0.003 to 7.144
Sex = 2	0 ^b^						
Age	0.029	0.0676	0.433	0.666	−0.105 to 0.163	1.030	0.901 to 1.177

Probability distribution: Multinomial; Link function: Cumulative logit; Target: Treatment Adherence; ^b^ This coefficient was set to zero because it is redundant.

**Table 8:** Socioeconomic Predictors of Adherence and Disease Activity.

Variable	Outcome	Statistic	*p*-Value
Education	Adherence	—	0.827
Occupation	Adherence	—	0.762
Occupation	DAS28 (RA)	F = 7.056	0.001
Occupation	ASDAS (AS)	F = 3.263	0.026

**Table 9:** Social Isolation and Quality of Life (GLMM).

Variable	OR	95% CI	*p*-Value
Social isolation	45.24	17.71–115.56	<0.001

**Table 10:** Communication and Anxiety.

Predictor	Outcome	OR	95% CI	*p*-Value
Better communication	Lower anxiety severity	2.19	1.57–3.07	<0.001
Better communication	Higher adherence	2.84	1.71–4.73	<0.001
Higher anxiety severity	Poorer adherence	6.40	2.08–19.65	0.001

**Table 11:** Hospitalization Duration.

Variable	F	*p*-Value
Communication	3.215	0.024
Dietary support	3.766	0.006
Adherence	2.092	0.084

**Table 12:** Costs and Inflammation.

Variable	β	F	*p*-Value
CRP	0.06	5.953	0.020
Time × CRP	−0.001	5.531	0.025
DAS28	—	—	0.140

## Data Availability

The original contributions presented in this study are included in the article. Further inquiries can be directed to the corresponding authors.

## Abbreviations

The following abbreviations are used in this manuscript:

AS	Ankylosing Spondylitis
ASDAS	Ankylosing Spondylitis Disease Activity Score
BASDAI	Bath Ankylosing Spondylitis Disease Activity Index
CRP	C-reactive Protein
DAS28	Disease Activity Score in 28 Joints
ESR	Erythrocyte Sedimentation Rate
GLMM	Generalized Linear Mixed Model
IQR	Interquartile Range
LMM	Linear Mixed Model
OR	Odds Ratio
RA	Rheumatoid Arthritis
QoL	Quality of Life
SD	Standard Deviation
